# DYSBIOSIS IN ACUTE-ON-CHRONIC LIVER FAILURE - FROM A PATHOPHYSIOLOGICAL COMPONENT TO A THERAPEUTIC TARGET

**DOI:** 10.1590/S0004-2803.24612025-141

**Published:** 2026-07-24

**Authors:** Angelo A. MATTOS, Cristiane A. ALVES, Johana ACUÑA, Angelo Z. MATTOS

**Affiliations:** 1 Universidade Federal de Ciências da Saúde de Porto Alegre, Programa de Pós-Graduação em Medicina: Hepatologia, Porto Alegre, RS, Brasil.; 2 Irmandade Santa Casa de Misericórdia de Porto Alegre, Unidade de Gastroenterologia e Hepatologia, Porto Alegre, RS, Brasil.

**Keywords:** Cirrhosis, acute decompensation, acute-on-chronic liver failure, microbiome, dysbiosis, Cirrose, descompensação aguda, insuficiência hepática aguda sobre crônica, microbioma, disbiose

## Abstract

**Background:**

Acute-on-chronic liver failure (ACLF) affects approximately one-third of patients hospitalized for acute decompensation of cirrhosis. These patients exhibit an extremely high degree of systemic inflammation, and infections as well as severe alcohol-related hepatitis are the most common precipitating factors of ACLF.

**Objective:**

This paper aims to discuss the most relevant aspects of ACLF emphasizing the role of gut dysbiosis.

**Methods:**

This review includes clinical and epidemiological studies, meta-analyses, and other articles published in English and indexed in the following databases: PubMed, Scopus, and Embase. Only full-text articles were selected.

**Results:**

ACLF is the most severe complication in patients with cirrhosis and is associated with high mortality rates. Bacterial translocation is considered responsible for the systemic inflammation leading to acute decompensation of cirrhosis when other precipitating events are not identified. Different microbiome profiles may influence the incidence of decompensation and thus the clinical course of the disease. Dysbiosis causes intestinal inflammation, which contributes to gut barrier dysfunction and pathological bacterial translocation, the main triggering factor of the cascade leading to acute decompensation of cirrhosis and multiple organ failure. Since dysbiosis plays a central role in the pathophysiology of acute decompensation of cirrhosis and ACLF, it is expected that treatments targeting the microbiome could modify the course of the disease. Despite current limitations, the role of probiotics, prebiotics, postbiotics, rifaximin, bacteriophages, and fecal microbiota transplantation is discussed in the present review.

**Conclusion:**

ACLF is a highly significant complication of liver disease. Dysbiosis and the gut-liver axis play key roles in its pathophysiology. This knowledge supports the idea that manipulation of the microbiome may be a potential therapeutic strategy.

## HIGHLIGHTS

Acute-on-chronic liver failure is the most severe form of acute decompensation of cirrhosis owing to its high mortality. It is present in more than 30% of hospitalized patients with cirrhosis, with bacterial infections being the most common precipitating factor. This article reviews the epidemiology, pathogenesis, and therapeutic alternatives for acute-on-chronic liver failure, emphasizing the role of gut dysbiosis.

## RESUMO

**Contexto:**

A insuficiência hepática aguda sobre crônica (ACLF) afeta aproximadamente um terço dos pacientes hospitalizados por descompensação aguda da cirrose. Esses pacientes apresentam um grau extremamente elevado de inflamação sistêmica, e as infecções, bem como a hepatite alcoólica grave, são os fatores precipitantes mais comuns de ACLF.

**Objetivo:**

Este trabalho tem como objetivo discutir os aspectos mais relevantes da ACLF, enfatizando o papel da disbiose intestinal.

**Métodos:**

Esta revisão incluiu estudos clínicos, epidemiológicos, metanálises e outros artigos disponíveis em inglês nas seguintes bases de dados: PubMed, Scopus e Embase. Apenas artigos completos foram selecionados.

**Resultados:**

A ACLF é a complicação mais grave em pacientes cirróticos e está associada a altas taxas de mortalidade. A translocação bacteriana é considerada responsável pela inflamação sistêmica que leva à descompensação aguda da cirrose quando outros eventos precipitantes não são identificados. Diferentes perfis de microbioma podem influenciar a incidência de descompensação e, consequentemente, o curso clínico da doença. A disbiose causa inflamação intestinal, o que contribui para a disfunção da barreira intestinal e a translocação bacteriana patológica, o principal fator desencadeante da cascata que leva à descompensação aguda da cirrose e à falência múltipla de órgãos. Como a disbiose desempenha um papel central na fisiopatologia da descompensação aguda da cirrose e da ACLF, espera-se que tratamentos direcionados ao microbioma possam modificar o curso da doença. A despeito de suas limitações, o papel do uso de prebióticos, probióticos, pós-bióticos, rifaximina, bacteriófagos e do transplante de microbiota fecal é discutido na presente revisão.

**Conclusão:**

A ACLF é uma complicação de grande relevância nas doenças hepáticas. A disbiose e o eixo intestino-fígado desempenham papéis fundamentais em sua fisiopatologia. Esse conhecimento permite considerar a manipulação do microbioma como uma potencial estratégia terapêutica.

## INTRODUCTION

Acute-on-chronic liver failure (ACLF) is at the extreme end of the spectrum of severity of acute decompensation of cirrhosis. It is defined as an acute decompensation of cirrhosis with at least one organ failure and a high short-term mortality rate^1^. The importance of this condition is associated not only with its poor prognosis, but also with its incidence. When using the definition of ACLF proposed by the European Foundation for the Study of Chronic Liver Failure Consortium, which has the best performance in predicting mortality^2^, a systematic review with meta-analysis demonstrated that 35% of individuals hospitalized for acute decompensation of cirrhosis fulfill the criteria for ACLF^3^. Despite its severity and high incidence, there is still no specific treatment for ACLF, which is why it is essential to increase medical knowledge about its pathophysiology and possible therapeutic targets.

Bacterial infections are known to be the most common precipitating factor of ACLF worldwide (35% of cases)^4^. However, this proportion may be even higher in certain geographic regions. In Latin America, for instance, infections are responsible for over 47% of ACLF cases^4^.

The human microbiome consists of 10 to 100 trillion bacteria, fungi, viruses, and archaea. Bacteria can be autochthonous (*firmicutes*, *bacteroidetes*, and *proteobacteria*) or non-autochthonous (gram-negative bacteria such as *escherichia coli*, *klebsiella pneumoniae*, among others). Dysbiosis refers to an imbalance of the microbiome, resulting from alterations in its composition and function, which, in patients with cirrhosis, may trigger complications of the disease^5^.

When assessing the gut-liver axis, it is important to consider that the liver is particularly sensitive to changes in the microbiome or in intestinal permeability. As the liver receives 75% of its blood supply from the intestine through the portal vein, several molecules capable of crossing the intestinal barrier are implicated in liver damage. Endotoxins such as lipopolysaccharides are examples of pathogen-associated molecular patterns (PAMPs) that can reach the liver, promoting, among other alterations, macrophage activation and probably liver fibrosis. On the other hand, the liver may affect the gut microbiome by decreasing the flow of bile acids. Bile acids have bactericidal properties and are also modulators of the farnesoid X receptor, which is crucial to the homeostasis of the intestinal barrier. If the integrity of the intestinal barrier is compromised, bacterial translocation may lead to a further increase in the release of PAMPs. Thus, the liver acts both as a target and as a regulator of the microbiome^6^ ^,^ ^7^.

### Pathophysiology of acute decompensation and acute-on-chronic liver failure

According to the systemic inflammation hypothesis, a pro-inflammatory and pro-oxidative status is deeply implicated in the pathophysiology of cirrhosis decompensation^8^. In this context, one could consider the existence of an “immune-inflammatory dysbiotic disorder”, a systemic consequence of cirrhosis caused by the breakdown of the homeostasis of the gut-liver axis. Once initiated, it leads to a vicious cycle of bacterial translocation and inflammation mediated by PAMPs and damage-associated molecular patterns (DAMPs - i.e., sterile inflammation), which assume an essential role in the development of the complications of the disease through further activation of the inflammatory cascade^9^. Among the complications of cirrhosis, the role of the microbiome has been most extensively studied in hepatic encephalopathy (HE)^10^.

In individuals with cirrhosis, the microbiome suffers from a reduced diversity of species, as well as changes in the predominant species (predominance of *fusobacteria*, *proteobacteria*, *enterococcaceae*, and *streptococcaceae*, with relative reductions in *bacteroidetes*, *ruminococcus*, *roseburia*, *veillonellaceae*, and *lachnospiraceae*). Potentially beneficial taxa, such as *akkermansia*, are found to be diminished in patients with different etiologies of liver disease. In addition, there is bacterial overgrowth in the small intestine, partly as a result of reduced intestinal motility^11^.

Acute decompensation of cirrhosis has recently been characterized to follow three different courses: stable acute decompensation, unstable acute decompensation, and pre-ACLF^12^. The two most severe forms of decompensation of cirrhosis, pre-ACLF and ACLF itself, are characterized by an extremely exacerbated state of systemic inflammation and are mostly precipitated by bacterial infection and/or severe alcohol-related hepatitis. Nevertheless, no precipitant is identified in approximately 30% of these patients, in whom bacterial translocation may be considered responsible for the systemic inflammatory state driving the decompensation of cirrhosis. Different microbiome profiles can influence the rate of decompensation and, therefore, the evolution of these patients^6^ ^,^ ^12^ ^,^ ^13^. In summary, dysbiosis causes intestinal inflammation, which, in turn, contributes to gut barrier dysfunction and pathological bacterial translocation, triggering the phenomena that lead to acute decompensation of cirrhosis and multiple organ failure^14^.

### Precipitating factors of acute-on-chronic liver failure

ACLF is a distinct clinical syndrome, not only because of its clinical features, but also because of the molecular characteristics related to its pathophysiology^14^. A more intense systemic inflammation is observed, indicated by significantly higher levels of leukocytes, C-reactive protein, and cytokines. Given the frequent inadequacy of blood biomarkers in assessing systemic inflammation, recent research has focused on quantifying it using gene expression profiles from circulating immune cells. This led to the creation of the CLIF-SIG score, a genetic score designed to quantify systemic inflammation in patients with acute decompensated cirrhosis. This score has demonstrated excellent predictive performance for the development of ACLF during hospitalization^15^. When evaluating the metabolomics of ACLF, patients show an increase in a wide variety of metabolites, some indicating inhibition of mitochondrial adenosine triphosphate (ATP) production in peripheral tissues, and others probably reflecting intestinal dysbiosis. The hypothesis of systemic inflammation finds its greatest clinical expression here^8^ ^,^ ^16^ ^,^ ^17^.

In the PREDICT study, which assessed the clinical courses of acute decompensation of cirrhosis in more than 1,500 patients, the importance of infection as a risk factor for ACLF was already highlighted, with spontaneous bacterial peritonitis (SBP) standing out^12^. This was followed by the PREDICT 2 study, which more specifically assessed the precipitating factors of acute decompensation of cirrhosis. When identifiable, the most prominent triggers of acute decompensation were bacterial infection (44% in individuals with ACLF and 22% in those with acute decompensation not fulfilling criteria for ACLF) and alcohol-related hepatitis (44% in ACLF and 19% in acute decompensation without ACLF)^13^. The importance of infections in this clinical scenario is also reinforced by a post-hoc analysis of the Global Study, which evaluated over 1,000 patients with decompensated cirrhosis in six regions of the world and found that bacterial infection was related to ACLF in 48% of cases, with a substantial role of infections caused by multidrug-resistant (MDR) bacteria^18^ ^,^ ^19^. Therefore, it could be advocated that prophylactic measures to prevent infections in patients with cirrhosis (including those caused by MDR bacteria) might prevent the development of ACLF.

Regarding alcohol-related hepatitis, alcohol consumption has direct and indirect effects on the gut microbiome, promoting changes in bacterial diversity and leading to bacterial overgrowth. However, it seems the interplay between alcohol and the gut microbiome might be bidirectional. There is evidence suggesting that the characteristics of the gut microbiome could predispose to behaviors usually linked to higher alcohol consumption and, consequently, increase the risk of developing alcohol-related liver disease. Therefore, there is recent interest in research on treatments that might modulate the gut microbiome, leading to a decrease in excessive alcohol consumption and ultimately reducing the risk of developing alcohol-related liver disease, or, when cirrhosis is already established, reducing the risk of acute decompensation and ACLF^20^.

### Antibiotic resistance

The use of antibiotics to modulate the gut microbiome and prevent SBP (and consequently to prevent ACLF) in patients with cirrhosis has been shown, when properly indicated, to reduce mortality^21^ ^-^ ^23^. Currently, however, it is critical to consider the challenge of antimicrobial resistance in cirrhosis. The World Health Organization (WHO) points to antimicrobial resistance as one of the greatest threats to global health. Infections associated with MDR bacteria cause approximately 1.14 million deaths per year worldwide and are expected to rise to 10 million by 2050 if no action is taken. The indiscriminate use of antimicrobials has driven the emergence of resistance, with reports of bacteria resistant to 60% of antimicrobials in some countries, which constitutes a real threat that antibiotics will no longer work in the future. It should be noted that patients with cirrhosis are at high risk of resistance: antibiotics are frequently prescribed (25% are long-acting antibiotics), and these individuals often undergo invasive procedures and have recurrent hospitalizations^24^ ^,^ ^25^.

It is also within this context that the finding of MDR bacteria colonization, observed by rectal swabs, increases the risk of infection by the colonizing strain in patients with cirrhosis. When two series of critically ill patients with cirrhosis were evaluated, more than 40% of patients were colonized by MDR bacteria. Interestingly, colonization by MDR microorganisms was also associated with an increased risk of infection by MDR bacteria, and infections occurring in carriers were caused mainly by the colonizing strain^26^.

Moreover, a metagenomic analysis of the gut microbiome of patients with cirrhosis has demonstrated that these patients have a high microbial gene load of antibiotic resistance genes compared to controls, which is related to the progression of the disease and is associated with a higher number of hospitalizations and a higher mortality rate^27^. Anaerobic commensal bacteria form an important reservoir of antibiotic resistance genes. Horizontal gene transfer due to conjugation, plasmid transfer, and bacteriophage-mediated transduction appears to be a common event, even among bacteria of the same phylum (such as *firmicutes*). Opportunistic pathogens, such as *enterobacteriaceae* and *enterococcaceae*, can also acquire resistance genes from the gut microbiome (at lower rates compared to commensal anaerobes). Resistance genes can cause phenotypic resistance through enzymatic inactivation, modification of the target of the antibiotics, or prevention of their intracellular accumulation through efflux pumps. The role of host-mediated conditions that can affect horizontal gene transfer is also recognized^28^. Therefore, acting on the gut microbiome to prevent and reduce resistance might have a significant impact on patient outcomes, as well as on reducing environmental contamination with MDR bacteria.

Some studies suggest that antibiotic prophylaxis could lead to an increase in bacterial resistance. Recently, two studies raised concerns about an increase in the incidence of SBP associated with the use of fluoroquinolones and sulfamethoxazole-trimethoprim. In the context of primary prophylaxis, an increase in bacterial resistance was observed, particularly associated with the use of fluoroquinolones, as well as a worse clinical course among individuals who were on antibiotic prophylaxis^29^. Similarly, in the context of secondary prophylaxis, a higher risk of infection recurrence was observed among individuals under antibiotic prophylaxis, a risk that was proportional to the length of antibiotic use^30^. On the other hand, other authors have shown that long-term antibiotic prophylaxis with fluoroquinolones was not associated with an increase in the presence of MDR bacteria^18^ ^,^ ^31^.

Considering the heterogeneity of results of the mentioned studies and the uncertainty regarding the safety of antibiotic prophylaxis, other forms of prophylaxis not using antibiotics might become attractive. A systematic review with meta-analysis of 13 controlled studies evaluated the role of probiotics in individuals with cirrhosis. In that study, the use of *bifidobacterium* with or without *lactobacillus* was associated with a reduction in ammonia levels and in the incidence of hepatic encephalopathy (similarly to lactulose), although it did not decrease the incidence of SBP or mortality^32^. Current evidence does not support significant changes in clinically relevant outcomes with the use of probiotics in chronic liver disease^7^. Lactulose acts as a prebiotic but does not seem to have a substantial impact on the gut microbiome. It accelerates intestinal transit and acidifies the intestinal lumen, thereby reducing ammonia production in the gut, increasing its fecal excretion, and decreasing its reabsorption^33^. There are also promising therapies using postbiotics, which are preparations of inanimate microorganisms and/or their components that could impart potential benefits to the health of the host^33^.

Yet another alternative to modulate the gut microbiome is the use of rifaximin. Rifaximin is a broad-spectrum non-absorbable antibiotic, which inhibits RNA-dependent polymerase and has a still not completely elucidated action in the management of hepatic encephalopathy. Partly, its action is related to bacteriophages that target *streptococcus* urease producers. It also has a eubiotic action by preserving beneficial bacterial taxa such as *lactobacillus* ^11^. Its role in decreasing the recurrence of hepatic encephalopathy was demonstrated in a seminal multicenter double-blind randomized controlled study published in 2010^34^. A recent randomized, double-blind, placebo-controlled trial evaluating rifaximin demonstrated no beneficial effect on 12-month survival or the incidence of complications of cirrhosis in patients with severe cirrhosis and low ascitic fluid protein levels^35^.

Proton-pump inhibitors may interfere with the gut microbiome by promoting enteric colonization, bacterial overgrowth, and bacterial translocation. Their use has been identified as a risk factor for the development of hepatic encephalopathy and infections, although it is controversial whether they actually increase the risk of SBP^36^ ^-^ ^38^. Therefore, the prescription of proton-pump inhibitors should be limited to strict indications.

### Future therapies

Phages (viruses that infect bacteria) are among the most promising therapies based on microbiome modulation. When phages recognize the receptor on the surface of the bacterial cell wall, they inject their DNA, which is replicated and occupies the structures of the bacterial wall. Afterwards, the enzymes of the phages promote the lysis of the bacterial wall, thus releasing the newly produced phages, which will maintain a self-perpetuating predatory relationship with the bacteria. Phages have been used for a long time as antimicrobial agents, but their use was progressively replaced by the prescription of antibiotics. Now, with the increase in the incidence of infections associated with MDR bacteria, the improvement in the comprehension of the human microbiome, and genome sequencing, phage-based therapy is gaining new interest. This therapy is considered safe, even if administered intravenously^33^.

Another interesting alternative is the use of genetic engineering. With the advent of synthetic biology, which combines the tools of molecular biology and the principles of advanced engineering, biologic circuits can be designed, allowing for advancement in microbial therapies, such as the use of genetic engineering to create bacteria with a potentially beneficial profile^39^.

Fecal microbiota transplant (FMT) has progressively gained more attention in different clinical scenarios. It was first performed in China over 1,500 years ago, and it began to be used in the West for the treatment of pseudomembranous colitis in 1958^40^, finally becoming a Food and Drug Administration-approved treatment for *C. difficile* infection in 2022^41^.

Evidence on the use of FMT in hepatology is still very limited, mostly from phase 1 and 2 studies and directed at patients with metabolic dysfunction-associated steatotic liver disease (MASLD), alcohol-related liver disease (ALD), and hepatic encephalopathy^7^. Further studies are necessary to investigate a possible role of FMT in preventing infection, which seems to be the most important trigger of ACLF.

In this context, in a phase 1 study with patients with advanced cirrhosis using lactulose and rifaximin, FMT restored the microbiome previously disrupted due to the use of antibiotics^42^. Another study analyzed data from 40 individuals with cirrhosis who underwent FMT and evaluated its impact on antibiotic resistance genes. It revealed that resistance was highly reduced by FMT both in comparison to baseline findings and when compared to controls^43^. Furthermore, a systematic review of five studies (52 patients) assessed the impact of FMT on intestinal bacterial colonization and resistance to antibiotics. Despite the low quality of the evidence, that systematic review demonstrated that FMT led to decolonization of MDR bacteria in half of the treated individuals, with a low rate of temporary adverse effects^44^.

Intestinal microbiome manipulation has also been the focus of research in the management not only of ALD, but also of alcohol use disorder itself. As previously mentioned, alcohol consumption has direct and indirect effects on the intestinal microbiome, since alcohol metabolism leads to the activation of an inflammatory cascade and changes in the peripheral nervous system. By modifying the gut microbiome, alcohol intake might be modulated, slowing ALD progression^20^. To achieve gut microbiome modulation, FMT has been studied, and there is initial evidence suggesting that FMT might reduce craving and alcohol intake^45^, as well as decrease the incidence of clinical complications (ascites, hepatic encephalopathy, infections, and hospitalizations) and increase survival^46^.

Moreover, FMT has recently been evaluated in the management of severe alcohol-related hepatitis, with encouraging results^47^. In a cohort of 33 patients with ACLF triggered by alcohol-related hepatitis, individuals who underwent FMT had higher 28- and 90-day survival rates, as well as better control of complications of cirrhosis (hepatic encephalopathy and ascites)^48^.

Despite being a promising therapy, safety concerns have been raised regarding the use of FMT in patients with decompensated cirrhosis. In a systematic review of two randomized controlled trials and three retrospective case series, FMT was associated with higher rates of severe adverse effects (including death) in this population. Therefore, at this time, the use of FMT in decompensated cirrhosis should be restricted to research^49^. The key points to consider when discussing FMT in cirrhosis are: the quality of the donor; the lack of efficacy of using antibiotics prior to the procedure (they are not only unnecessary, but can also be damaging); the possibility that changes in microbial function are more relevant than those in microbial structure; and the lack of knowledge on ideal doses, frequency, and routes of administration^10^.

## CONCLUSION

Given the growing understanding of the role of dysbiosis and the gut-liver axis in the pathophysiology of liver diseases and, particularly, in the development of acute decompensation of cirrhosis and ACLF, turning the manipulation of the microbiome into a therapeutic target seems extremely appealing. Therefore, the use of prebiotics, probiotics, postbiotics, antibiotics, bacteriophages, and even FMT could become part of the management of cirrhosis in the medium-term future.

## Data Availability

Not applicable
